# Cross-Sectional Characterization of Local Brain Network Connectivity Pre and Post Breast Cancer Treatment and Distinct Association With Subjective Cognitive and Psychological Function

**DOI:** 10.3389/fneur.2021.746493

**Published:** 2021-10-29

**Authors:** Shelli R. Kesler, Tien Tang, Ashley M. Henneghan, Michelle Wright, M. Waleed Gaber, Oxana Palesh

**Affiliations:** ^1^School of Nursing, University of Texas at Austin, Austin, TX, United States; ^2^Department of Pediatrics, Baylor College of Medicine, Houston, TX, United States; ^3^Department of Psychiatry and Behavioral Sciences, Stanford University, Palo Alto, CA, United States

**Keywords:** breast cancer, fMRI, cognition, effective connectivity, functional connectivity, Bayesian network, graph theory

## Abstract

**Objective:** We aimed to characterize local brain network connectivity in long-term breast cancer survivors compared to newly diagnosed patients.

**Methods:** Functional magnetic resonance imaging (fMRI) and subjective cognitive and psychological function data were obtained from a group of 76 newly diagnosed, pre-treatment female patients with breast cancer (mean age 57 ± 7 years) and a separate group of 80, post-treatment, female breast cancer survivors (mean age 58 ± 8; mean time since treatment 44 ± 43 months). The network-based statistic (NBS) was used to compare connectivity of local brain edges between groups. Hubs were defined as nodes with connectivity indices one standard deviation or more above network mean and were further classified as provincial (higher intra-subnetwork connectivity) or connector (higher inter-subnetwork connectivity) using the participation coefficient. We determined the hub status of nodes encompassing significantly different edges and correlated the centralities of edges with behavioral measures.

**Results:** The post-treatment group demonstrated significantly lower subjective cognitive function (W = 3,856, *p* = 0.004) but there were no group differences in psychological distress (W = 2,866, *p* = 0.627). NBS indicated significantly altered connectivity (*p* < 0.042, corrected) in the post-treatment group compared to the pre-treatment group largely in temporal, frontal-temporal and temporal-parietal areas. The majority of the regions projecting these connections (78%) met criteria for hub status and significantly less of these hubs were connectors in the post-treatment group (*z* = 1.85, *p* = 0.031). Subjective cognitive function and psychological distress were correlated with largely non-overlapping edges in the post-treatment group (*p* < 0.05).

**Conclusion:** Widespread functional network alterations are evident in long-term survivors of breast cancer compared to newly diagnosed patients. We also demonstrated that there are both overlapping and unique brain network signatures for subjective cognitive function vs. psychological distress.

## Introduction

Cancer and its treatments are associated with risk for cognitive dysfunction, most commonly in the domains of executive functioning, memory, attention, and processing speed (1, 2). Neuroimaging studies suggest that cancer-related cognitive impairment (CRCI) results from injury to brain structure and function. We previously showed that breast cancer survivors have altered brain network connectivity compared to healthy controls and chemotherapy naïve survivors (3–9). Other groups have subsequently observed similar findings (10–20).

Most of these studies, including our own, have focused largely on global brain network characteristics and/or local connectivity among a limited number of discrete regions. The advantages of these approaches include the provision of single, parsimonious metrics that summarize the vast complexity of the entire brain network (global properties) and the testing of specific hypotheses (limited local connectivity). Examination of all potential differences among local brain network connections is agnostic and requires larger samples but can provide a broader profile of potential biomarker features for modeling applications.

For example, we have previously utilized brain connectivity (connectome) features in machine learning models for predicting future cognitive outcome following breast cancer chemotherapy treatment (21, 22) and identifying neurophysiological clusters associated with cognitive impairment subtypes (8). However, these required complex feature selection/dimension reduction techniques due to limited *a priori* data regarding important local features. Additionally, there are emerging methods for deriving transcriptome profiles of connectome topology (23–25). These methods integrate region-specific transcriptomes with regional connectome properties (24). For example, Zhao et al. (26) used transcriptome-connectome correlation analysis to identify gene signatures of age-related brain functions. Transcriptome methods could provide unique insights regarding the genetic and epigenetic mechanisms underlying CRCI.

In terms of additional molecular mechanisms, our preclinical studies indicated that chemotherapy treatment leads to significant mitochondrial damage resulting in decreased respiratory activity (27). Hubs are highly connected regions responsible for the majority of information processing and exchange (28, 29). As a result, they require much greater metabolic resources than non-hub regions (30–32). Hubs are believed to play a role in the transmission of neuropathology (33) as well as the maintenance of brain network resilience (34). Our prior studies have examined hub status within the global network but not within local networks (3, 4, 21). Few other studies have examined hub status and these have also focused exclusively on the global network (13, 35).

One of the difficulties in clinical management of CRCI is disentangling cognitive effects from psychological distress and fatigue. Since subjective cognitive outcomes are typically correlated with distress while objective outcomes are not (36), clinicians and researchers often conclude that objective measures reflect neuropathology while subjective reports represent distress. However, there is limited empirical evidence to support this theory. On the contrary, several studies, including our own have shown that self-report measures of cognitive function do correlate with neuroimaging metrics (22, 37–39), suggesting that subjective cognitive impairments may represent a unique phenotype of CRCI. Distress is more often “controlled” statistically in neuroimaging studies of CRCI, leaving gaps in knowledge concerning the neural underpinnings of distress within the context of CRCI.

In this study we aimed to compare local functional brain connectivity in patients seen pre-treatment with those seen post-treatment. This included determining the hub status of local connectivity differences and examining local connectivity profiles associated with subjective cognitive function and psychological distress.

## Methods

### Participants

In this retrospective study, we examined neuroimaging and self-rating questionnaire data that was commonly acquired across all participants. These included 76 newly diagnosed female patients with breast cancer and 80 female breast cancer survivors. Participants were age 43-81 years, newly diagnosed patients were evaluated prior to primary cancer treatment (surgery with general anesthesia, chemotherapy, radiation) and survivors were evaluated at least 6 months post primary cancer treatment (Table 1) in order to allow for medical stabilization and to focus on patients who may have longer-term, more persistent cognitive deficits. Newly diagnosed patients (PRE-TX group) were assessed between 2012 and 2020 and survivors (POST-TX group) were assessed between 2008 and 2016, all at a single university. This study was approved by the Stanford University Institutional Review Board, was conducted in accordance with the ethical standards of the Declaration of Helsinki and all participants provided written informed consent.

**Table 1:** Participant characteristics and behavioral data.

	**PRE-TX**	**POST-TX**	**Statistic**	***P*-value**
Age (years)	56.7 (7.1)	58.6 (8.0)	t = 1.50	0.135
Age range	44-75	43-81		
Education (years)	15.8 (2.7)	16.2 (2.5)	t = 0.934	0.352
Time since primary treatment (months)	-	44.3 (43.5)range: 6-157	-	-
Radiation	-	78%	-	-
Hormone blockade	-	67%	-	-
Chemotherapy	-	100%	-	-
Behavioral rating inventory of executive function adult (BRIEF-A)	51.4 (9.3)	59.8 (16.5)	W = 3856	0.004
BRIEF-A clinically significant	0%	38%	z = 5.94	<0.001
Clinical assessment of depression (CAD)	51.4 (9.8)	50.3 (13.9)	W = 2866	0.627
CAD clinically significant	5%	9%	z = 1.02	0.154

*Values are shown as mean (standard deviation) unless otherwise noted*.

### Self-Report Data

Self-ratings of psychological function were obtained using the Total Score of the Clinical Assessment of Depression (CAD), which measures, depression, anxiety and fatigue (40). Subjective executive function was measured with the Global Executive Score of the Behavioral Rating Inventory of Executive Function-Adult Version (BRIEF-A) (41). Raw scores were converted to T scores (mean = 50 ± 10) based on the published normative data for each test. The clinical cutoff score for BRIEF-A is 65 (41) and is 69 for CAD (40). Participants also completed objective cognitive testing but these measures were different for PRE vs. POST participants and therefore could not be combined.

### Neuroimaging Acquisitions

Functional magnetic resonance imaging (fMRI) data were obtained while participants rested with eyes closed using a T2^*^ weighted (42) gradient echo spiral pulse sequence: TR = 2,000 ms, TE = 30 ms, flip angle = 80° and 1 interleave, FOV = 22 cm, matrix = 64 x 64, in-plane resolution = 3.4375, number of volumes = 216, oblique prescription with a 3T GE Signa HDx whole body scanner (GE Medical Systems, Milwaukee, WI). A high-order shimming method was employed to reduce field heterogeneity. A high-resolution, 3D IR prepared FSPGR scan was also acquired and used for spatial normalization of fMRI: TR: 8.5, TE: minimum, flip: 15°, TI: 400 ms, BW: ±31.25 kHz, FOV: 22 cm, Phase FOV: 0.75, slice thickness: 1.5 mm, 124 slices, 256 x 256 @ 1 NEX, scan time: 4:33. Data were visually inspected for quality.

### Neuroimaging Preprocessing

Resting state fMRI were preprocessed with Statistical Parametric Mapping 12 and CONN Toolboxes (43, 44) implemented in Matlab v2019b (Mathworks, Inc, Natick, MA). Briefly, this involved realignment, coregistration with the segmented anatomic volume, spatial normalization, and artifact detection followed by band-pass filtering (0.008-0.09 Hz). The CompCor correction method was used to reduce physiological and other non-neuronal noise artifacts (45). Motion parameters from realignment were included as regressors and images identified as motion or signal outliers using Artifact Detection Tools (46) were excluded (global signal = 3.0 standard deviations, motion = 1.0 mm, rotation = 0.05 mm). We determined *a priori* that participants with more than 10% outlier volumes would be excluded from the analysis though none met this threshold. Temporal correlations between all possible pairs of 90 cortical and subcortical bilateral regions (47) were computed based on the mean corrected fMRI signal. This resulted in a 90 x 90 functional connectome matrix for each participant where regions were defined as nodes and the temporal correlations between nodes were defined as edges (48).

### Statistical Analyses

We compared BRIEF-A and CAD scores between groups using two-tailed Wilcoxon rank-sum tests given inhomogeneity of variance. We also examined the difference in frequency of clinically significant scores (based on published cutoff scores) using a two-tailed z test for proportions.

We compared local connectivity within connectomes using the Network-based Statistic (NBS) Toolbox v1.2 (49), which identifies connected substructures, or components, within the larger network, similar to the cluster-based thresholding approach used in traditional voxel-wise neuroimaging analyses (49). Permutation testing with 5,000 permutations was then used to determine edge differences in components between the groups, controlling for multiple comparisons using family-wise error (FWE) and covarying for age. NBS results were visualized using BrainNet Viewer (50).

Brain regions identified as having significant NBS differences between groups were then evaluated for network hub status based on degree, betweenness centrality and/or clustering coefficient > 1 standard deviation above network mean (51). Hubs were further classified as provincial or connector type based on module participation coefficient. First, modularity analysis was conducted at minimum connection density across groups, which refers to the highest threshold where all nodes remain connected (no isolated nodes) (52). Modularity was calculated using the method described by Newman (53) and module decomposition was visually inspected for quality assurance. Hubs with module participation coefficient *P* < 0.3 were classified as provincial hubs, and hubs with *P* > 0.3 were classified as connector hubs (51). Hub results were visualized using BrainNet Viewer (50).

To explore relationships between connectome edges and self-rating scores, we first calculated the edge betweenness centrality for each edge identified as significantly different between groups by NBS. Betweenness centrality indicates the importance of each edge for the network's integration (54). We calculated centrality at the minimum connection density across groups. We then correlated the centralities with BRIEF-A and CAD scores using Spearman two-tailed correlations (*p* < 0.05) in the POST-TX group only given that BRIEF-A scores were significantly higher in this group (indicating greater executive dysfunction). Given that these were exploratory correlations, we did not correct for multiple comparisons. We also correlated BRIEF-A and CAD scores within the POST-TX group.

## Results

The POST-TX group demonstrated significantly greater executive dysfunction as measured by the BRIEF-A (*W* = 3,856, *p* = 0.004) but the groups did not differ in terms of CAD scores (*W* = 2,866, *p* = 0.627, Table 1). Further, there were 30 participants in the POST-TX group (38%) who had clinically significant BRIEF-A scores compared to 0 (0%) in the PRE-TX group, which was a significant difference (*z* = 5.94, *p* < 0.001). There were 7 (9%) participants in the POST-TX group with clinically significant CAD scores compared to 4 (5%) in the PRE-TX group. This was not a significant difference (*z* = 1.02, *p* = 0.154).

**Figure 1:**
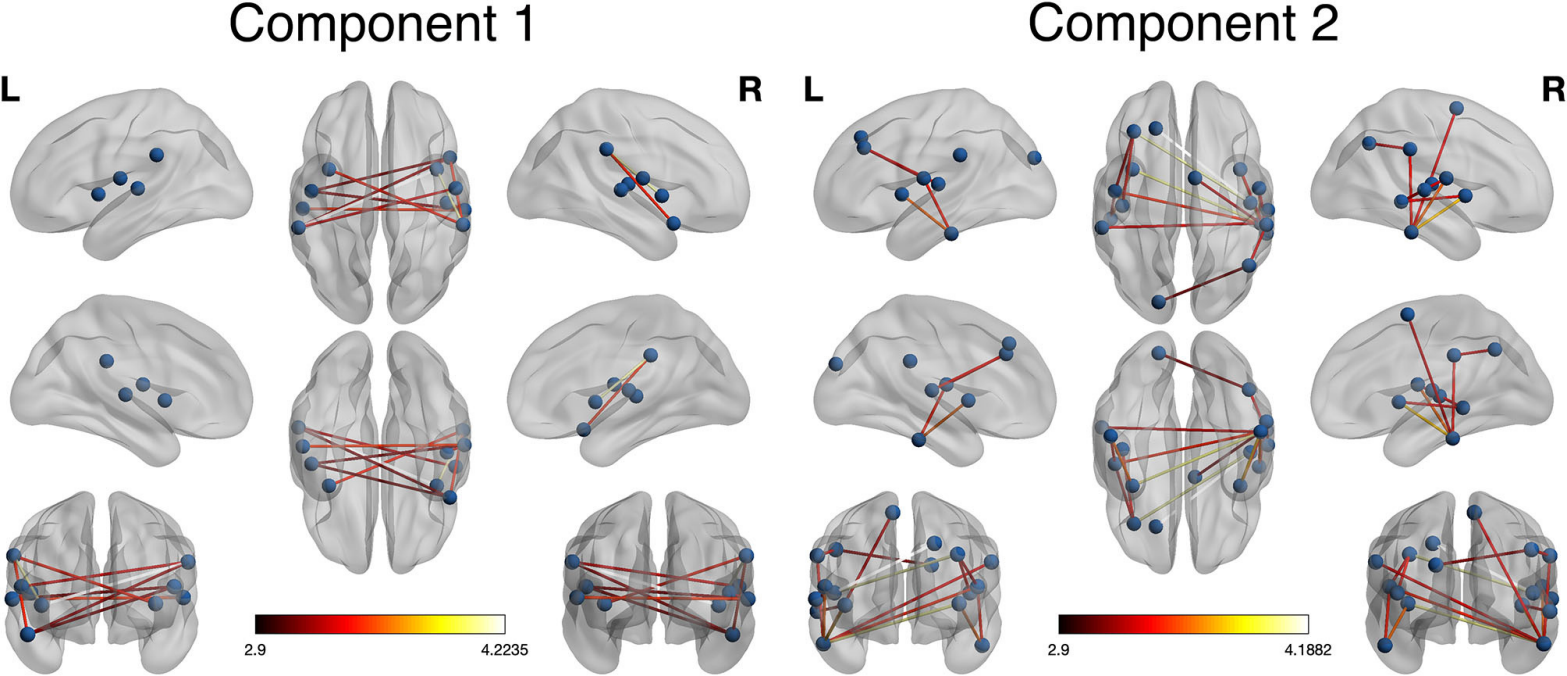
Network-based statistic (NBS) visualization. Two components were identified by NBS that differed significantly between groups. Component 1 included connections among lateral temporal and parietal regions. These connections were hypo-connected in the POST-TX compared to the PRE-TX group (colorbar indicates *t* statistic, *p* = 0.042, FWE corrected). Component 2 included connections in the frontal, temporal, parietal, and occipital regions. These connections were hyper-connected in the POST-TX group (colorbar indicates *t* statistic, *p* = 0.006, FWE corrected).

As shown in Figure 1, NBS revealed that two main network components were significantly different between the groups. Component 1 was hypo-connected in the POST-TX group and consisted of edges connecting left supramarginal gyrus with right insula and right Rolandic operculum, right supramarginal gyrus with bilateral insula, right superior temporal pole with left Rolandic operculum and bilateral supramarginal gyrus, and right superior temporal gyrus with right Heschl's gyrus, left Rolandic operculum and left superior temporal gyrus (10 edges). The t statistics for these edges ranged from 3.11 to 4.22 (*p* < 0.042, FWE corrected, Table 2).

**Table 2:** Network-based statistic results.

**Component 1**	***P*** **=** **0.042 FWE corrected**		**Component 2**	***P*** **=** **0.006 FWE corrected**	
**Region 1**	**Region 2**	**T score**	**Region 1**	**Region 2**	**T score**
Left rolandic operculum	Right superior temporal pole	3.11	Left middle frontal	Left superior occipital	3.11
Left rolandic operculum	Right superior temporal	3.13	Left superior frontal	Right superior temporal	3.11
Left supramarginal	Right superior temporal pole	3.15	Left middle frontal	Left rolandic operculum	3.25
Right rolandic operculum	Left supramarginal	3.26	Right angular	Right inferior temporal	3.28
Right Heschl	Right superior temporal	3.4	Left middle frontal	Right middle temporal	3.28
Left insula	Right supramarginal	3.43	Right angular	Right inferior temporal	3.30
Right supramarginal	Right superior temporal pole	3.45	Left insula	Right middle temporal	3.31
Left superior temporal	Right superior temporal	3.47	Left rolandic operculum	Right supramarginal	3.32
Right insula	Right supramarginal	4.12	Left insula	Right Heschl	3.38
Right insula	Left supramarginal	4.22	Right insula	Right inferior temporal	3.38
			Left rolandic operculum	Left inferior temporal	3.41
			Right rolandic operculum	Right inferior temporal	3.43
			Right supplementary motor area	Left inferior temporal	3.55
			Left supramarginal	Right inferior temporal	3.55
			Right supramarginal	Right inferior temporal	3.74
			Right insula	Right inferior temporal	4.05
			Right rolandic operculum	Left Heschl	4.09
			Right middle temporal	Left Heschl	4.19

Component 2 was hyper-connected in the POST-TX group and included edges connecting left Heschl's gyrus with left middle and superior frontal gyri, right Heschl's with left middle frontal gyrus, left superior occipital gyrus with right angular gyrus, left Rolandic operculum with left middle frontal gyrus, right supramarginal gyrus with right angular gyrus, left inferior temporal gyrus with left insula and left Rolandic operculum, right inferior temporal gyrus with bilateral insula, bilateral Rolandic operculum, right supplementary motor area and bilateral supramarginal gyri, right middle temporal gyrus with right insula, right Rolandic operculum and right superior temporal gyrus with right middle temporal gyrus (18 edges). The t statistics ranged from 3.11 to 4.19 (*p* = 0.006, FWE corrected, Table 2).

**Figure 2:**
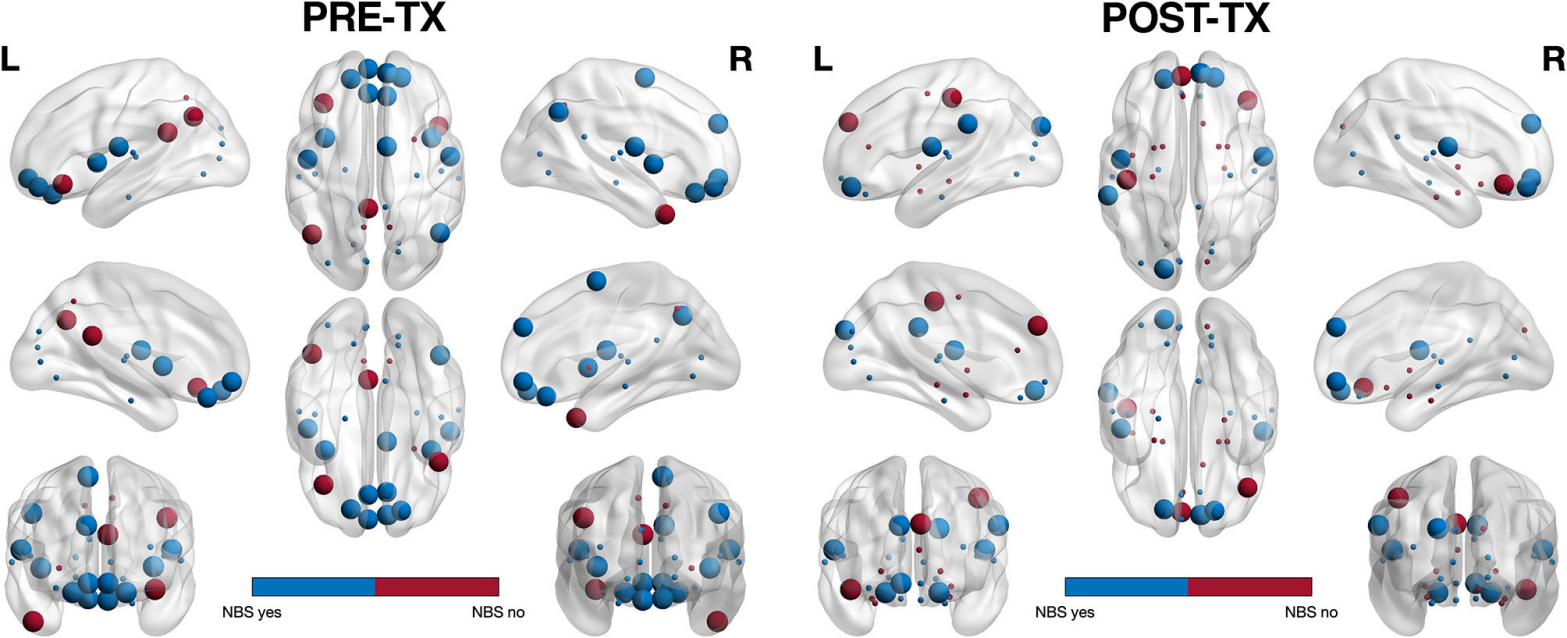
Hub profile visualization. Of the regions connected by NBS significant edges (shown in blue), 78% were classified as hubs. In the PRE-TX group, 52% of hub regions were connector (larger spheres) vs. provincial (smaller spheres) whereas in the POST-TX group, 70% were provincial. This difference was significant (*z* = 1.85, *p* = 0.031).

Most (78%) of the nodes that these altered edges connected were classified as hubs and 52% of these hubs were connector type, connecting to other functional subnetworks, in the PRE-TX group compared to 30% in the POST-TX group (Figure 2) (79). This difference was significant (z = 1.85, *p* = 0.031).

**Figure 3:**
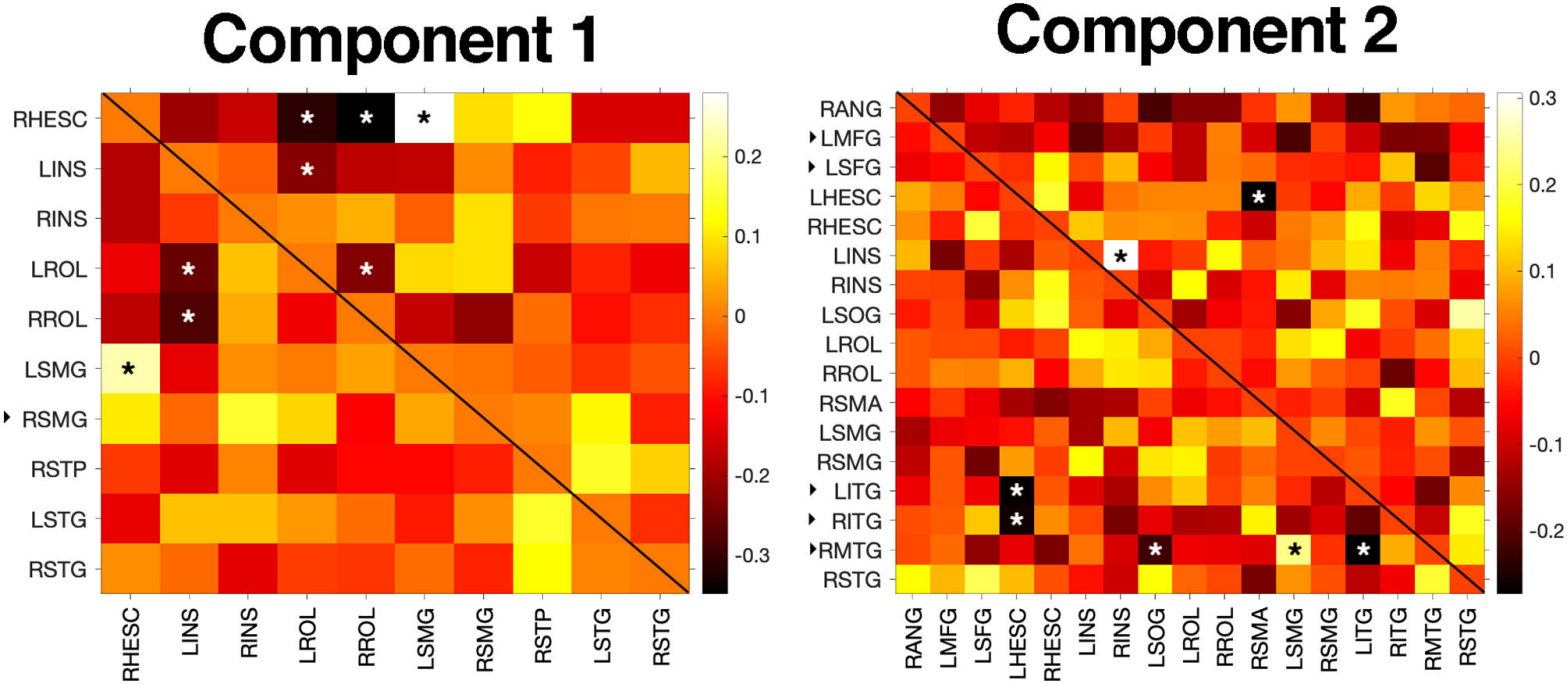
Correlation results. BRIEF-A and CAD scores were not correlated with centralities of significant NBS edges but were associated with centralities of several alternate edges connecting significant NBS nodes (*p* < 0.05). In the heatmaps, significant correlations with CAD are denoted with an asterisk in the upper triangle while correlations with BRIEF-A are denoted in the lower triangle. Colorbar indicates correlation coefficient. The black, right facing triangle next to a node label indicates a non-hub. All other nodes were hubs. L/RHESC, right Heschl's gyrus; L/RINS, left/right insula; L/RROL, left/right Rolandic operculum; L/RSMG, left/right supramarginal gyrus; RSTP, right superior temporal pole; L/RSTG, left/right superior temporal gyrus; RANG, right angular gyrus; LMFG, left middle frontal gyrus; LSFG, left superior frontal gyrus; LSOG, left superior occipital gyrus; RSMA, right supplementary motor area; L/RITG, left/right inferior temporal gyrus; RMTG, right middle temporal gyrus; RSTG, right superior temporal gyrus.

BRIEF-A and CAD scores were significantly correlated in the POST-TX group (*r* = 0.52, *p* < 0.001). Centralities for the significant NBS edges were not correlated with BRIEF-A or CAD scores in the POST-TX group. However, given that brain function tends to be non-linear and hierarchical, we further explored correlations among all edges for the significant NBS matrices (i.e., alternate connections between nodes encompassing significant NBS edges). For component 1, CAD correlated with five edges (*p* < 0.050, uncorrected) and BRIEF-A with three edges (*p* < 0.041, uncorrected) and two of these edges overlapped (i.e., were correlated with both scores). Component 2 showed the opposite profile with five edges correlating with BRIEF-A (*p* < 0.043, uncorrected) and two with CAD (*p* < 0.022, uncorrected) with no overlap (Figure 3).

## Discussion

Compared to the PRE-TX group, POST-TX breast cancer survivors demonstrated significantly altered local functional brain network connectivity. Specifically, our findings indicated group differences in the functional connections within temporal regions and among temporal-parietal and frontal-temporal regions. Since this study focused on connectivity, these findings suggest that information exchange between these regions may be altered following breast cancer treatment. Connections between temporal pole and supramarginal gyrus, as well as those between temporal pole and insula have been shown to be associated with word comprehension (55). Word comprehension *per se* has not been studied among breast cancer survivors, although potentially related problems such as difficulties with reading have been reported (56).

Inferior temporal gyrus is connected to supramarginal and angular gyri by inferior longitudinal fasciculus (ILF) and arcuate fasciculus (AF). AF connects frontal, parietal and temporal areas and is believed to be involved in language, praxis and visual spatial processing (57). ILF also connects to hippocampus, amygdala and cingulate to subserve vision, memory, emotion and language including the ventral-semantic stream (57). Insula connections with other temporal areas, including Heschl's gyrus, tend to be involved in cognition, emotion and interoception (58). Left middle and left superior frontal connections with left Rolandic operculum, left superior occipital, right superior temporal and right middle temporal regions likely involve attention and executive function integration with verbal memory, selective visual attention and somatosensory processing (59). Taken together, these findings may reflect the deficits in verbal memory, verbal fluency, attention, executive function and emotion regulation that are frequently observed among breast cancer patients and survivors (60–62).

Our findings may help direct future studies involving predictive modeling. A critical aspect of CRCI management is determining which patients are at highest risk. Neuroimaging data tend to be uniquely accurate in predicting future clinical and behavioral outcomes (63) but involve high dimensionality. Depending on the image resolution and parcellation scheme, neuroimaging data can provide potential features numbering in the tens of thousands or more. This is impractical for many clinical studies involving modest sample sizes. Therefore, findings such as ours can provide a smaller set of potential features for training predictive models. Similarly, a limited set of regions of interest would make interrogating cortical gene expression profiles more feasible, especially in a smaller sample (64).

Such studies involve examining the spatial correlations between brain-wide messenger RNA expression and regional neuroimaging metrics (25). Although many studies have demonstrated that primary breast cancer and/or its treatments significantly alter brain function and structure, it remains unclear how or why. Several studies, including our own, suggest genetic and/or epigenetic risk factors may play an important role (9, 39, 65–69). However, these findings have been limited to the candidate variants selected and have shown some inconsistent results, which may relate to what brain regions were examined. Regions of altered connectivity associated with CRCI could be correlated with an existing transcriptional atlas, such as the Allen Human Brain Atlas (70), to determine the gene expression profiles of those regions. This would provide more specific insights regarding the molecular mechanisms underlying altered connectivity profiles compared to the candidate genetic variant studies conducted thus far.

Most of the nodes that encompassed the altered edges in the POST-TX group were hub regions. This is consistent with prior literature suggesting that hub areas of the brain tend to be more vulnerable to injury and disease (32). Hubs have high activity and metabolism and are therefore believed to play a critical role in neurodegeneration. For example, amyloid-beta deposition in Alzheimer's disease has been shown to be associated with cortical hub connectivity (71). We have previously shown that connectivity of default mode network hubs can accurately distinguish chemotherapy treated breast cancer survivors from chemotherapy naïve survivors and healthy controls, suggesting that hubs may be preferentially vulnerable to chemotherapy (38). Our present results further indicate hub vulnerability across different subnetworks. Specifically, the hubs identified here are members of various functional subnetworks including default mode but also dorsal-attention and salience networks (72).

Further, the POST-TX group had significantly fewer connector hubs compared to the PRE-TX group. Connector hubs connect functional subnetworks to each other and are thus important for integrating information processing across the brain. Provincial hubs have higher intra-subnetwork connectivity and are therefore more important for local information exchange, or segregation (51). Lesioning of connector hubs tends to have a more widespread effect on brain network organization and efficiency (51, 73). Therefore, a greater proportion of provincial hubs following breast cancer therapy may reflect reorganization of neural resources to protect local processing in response to a reduction in global integration. Since cognitive domains such as executive function rely on interactions among various, anatomically distant regions, this may help explain the greater executive dysfunction endorsed by the POST-TX group. In fact, BRIEF-A scores were significantly correlated with the centralities of several edges and all of these edges connected one or more hubs (Figure 3).

BRIEF-A and CAD scores were significantly correlated with each other in the POST-TX group, consistent with prior reports (36, 74). However, BRIEF-A scores indicated significantly higher subjective executive dysfunction in the POST-TX group despite no significant difference in psychological distress. These results suggest that self-reported cognitive dysfunction cannot be sufficiently explained by distress. BRIEF-A and CAD scores did not correlate with the significant NBS edges. However, the non-linear nature of brain function (75, 76) in combination with potential brain network reorganization following injury can result in indirect effects of altered connectivity on clinical and behavioral outcomes. Accordingly, we found that centralities for alternate edges in the same component matrices were correlated with both BRIEF-A and CAD scores in the POST-TX group. Interestingly, there was limited overlap in these correlations. These results suggest that psychological distress and subjective cognitive dysfunction may have unique neurophenotypes.

However, correlations were exploratory and not corrected for multiple comparisons. Therefore, these findings should be interpreted with caution and further research regarding the neural correlates of subjective cognitive function vs. distress is warranted.

The present study is not without limitations including the cross-sectional design. Consistent with other such studies in this field, the POST-TX group was heterogenous for treatment regimen and there were not sufficient subsample sizes to examine effects of individual treatments. While longitudinal studies are optimal for examining individual cognitive trajectories and evaluating the effects of specific treatments, “broad” studies with larger samples are useful for identifying neuropsychiatric phenotypes (8, 77). Our study was also limited by its retrospective nature in terms of data availability. Objective cognitive function may have distinct neural correlates from those of subjective cognitive function. We did not have sufficient overlap in objective testing batteries for our retrospective cohorts to evaluate these effects. However, research regarding the neural correlates of subjective cognitive impairment has been very limited to date and therefore our findings make a significant contribution to this literature. The BRIEF-A measures executive function, which is very commonly affected by breast cancer and its therapies but the ability to assess other subjective cognitive domains would have strengthened our results. There was large variability in time since primary treatment completion of participants in the POST-TX group and a majority were undergoing hormone blockade therapy during this interval. It is possible that network structure changes over time across the post-treatment phase. Also, the rate of recovery after treatment could potentially alter hub status as hub profiles are dynamic. Therefore, assessment at multiple time points following breast cancer treatment could provide additional insights (21, 78).

Despite these limitations, our results provide further support that breast cancer and/or its treatments are associated with brain network alterations. This work provides further insights regarding the neural correlates of common cognitive-behavioral deficits observed in breast cancer survivors. These results could potentially inform predictive modeling applications and/or transcriptome studies, among others. Our findings also uniquely add to the growing body of work that patient reported outcomes are linked to measurable biomarkers. Our results suggest that self-reported CRCI and psychological symptoms may have distinct neurophenotypes, further indicating that CRCI and distress can co-occur and be jointly caused by post-treatment brain injury. This evidence includes guidance for future investigations of and potential interventions for subjective CRCI.

## Data Availability Statement

The raw data supporting the conclusions of this article will be made available by the authors, without undue reservation.

## Ethics Statement

The studies involving human participants were reviewed and approved by the Stanford University Institutional Review Board. The patients/participants provided their written informed consent to participate in this study.

## Author Contributions

SRK and OP designed and conducted the study. SRK conducted data analyses. SRK, TT, and AMH wrote the manuscript. SRK, MW, MWG, and OP reviewed/edited the manuscript. All authors contributed to the article and approved the submitted version.

## Funding

This research was supported by funding from the National Institutes of Health (R01CA226090 and R01CA172145 to SRK/OP and K01NR018970 to AMH).

## Conflict of Interest

The authors declare that the research was conducted in the absence of any commercial or financial relationships that could be construed as a potential conflict of interest.

## Publisher's Note

All claims expressed in this article are solely those of the authors and do not necessarily represent those of their affiliated organizations, or those of the publisher, the editors and the reviewers. Any product that may be evaluated in this article, or claim that may be made by its manufacturer, is not guaranteed or endorsed by the publisher.
